# Lice, Humans, and Microbes

**Published:** 2018-09

**Authors:** Saied Reza Naddaf

**Affiliations:** Department of Parasitology, Pasteur Institute of Iran, Tehran, Iran

Lice are small, wingless, minor ectoparasites of mammals and birds. More than 540 blood-sucking lice (Phthiraptera: Anoplura) have been described with each host having its own type of louse, suggesting the cospeciation of the lice species with their host. Among these, two lice species from two different genera infest humans: *Pediculus humanus* and *Phthirus pubis* (pubic “crab” lice). The former constitutes two morphotypes, *P. humanus* morphotype capitis (head lice) and *P. humanus* morphotype corporis (clothing “body” lice). Head, body, and pubic lice live on the head, in clothing, and in the pubic areas, respectively. These tiny creatures have been humans’ and primates’ close companions for millions of years and have played a significant role in human history, as they became the source of inspiration for many novelists. Human lice cannot survive on their target hosts for extended periods of time and die of starvation within 24-48 h. However, recent evidence implies human lice can shift hosts and adapt to other closely related species to evade death. This “host infidelity” has enabled these obligate, host-specific creatures to possibly survive the potential extinction of their host. This phenomenon has shed light on a novel way for scientists to study the history of human evolution, which previously relied on the fossil records, the human genome, and archaeological findings. One uncomfortable news came when DNA analysis of lice in human and other primates indicated acquisition of the pubic louse from gorilla by humans some 3.3 million years ago. This transfer might have happened when a group of human ancestors called *Australopithecus*, who represent the famous fossil Lucy, were around. Since infestation occurs in the pubic areas, the parasite transfer to early humans might have occurred during sexual contact, or by living near gorillas, perhaps by using the animals’ sleeping sites. DNA analysis of the human lice, *P. humanus*, from different areas, revealed a high sequence divergence and various clades, an indication for their evolution in the association with different archaic hominids. The distribution patterns of lice lineages in different areas suggest sympatry of *Homo sapiens* with other hominids in Asia and Europe, shaping a hypothesis on the acquisition of these lice lineages by modern humans from other archaic hominids like *Homo erectus*, Neanderthals and Denisovan men after they died out. The human lice studies also revealed when modern humans began wearing clothes. The molecular divergence of body lice from human head lice dates back to some 170,000 years ago when modern humans started wearing clothing and lice found themselves a suitable alternative niche to start diverging. Development of clothing, therefore, may have been an essential factor in the expansion of modern humans out of Africa.

“The history of lice and men, as Emily Willingham quotes, is a long, itchy story that intertwines co-evolution not only with these blood-sucking little parasites but also with the microbes they carry.” Of the three types of *lice* that live on humans, only *body lice* act as vectors of several life-threatening infectious diseases, which include epidemic typhus, trench fever, and relapsing fever, which are caused by *Rickettsia prowazekii, Bartonella quintana*, and *Borrelia recurrentis*, respectively. These infections typically occur during wars, social disruptions, and in areas where poor-hygiene conditions favor a high incidence of louse infestations. Humans are thought to be the unique reservoir of these agents. However, in the United States, China, and Japan, *B. quintana has been isolated from* other non-human primates. For instance, in the United States, the flying squirrel (*Glaucomys volans*) was identified as the reservoir of *R. prowazekii* in nature, causing some typhus cases in which human lice was not involved. *B. recurrentis* is an exception to borreliae, as it is the only relapsing fever species transmitted by *lice*. It appears to be a louse-adapted variant of the East African tick-borne *Borrelia duttonii*, with some degradation in genome size. Louse-borne relapsing fever (LBRF) is endemic in East Africa, but it might spread to other areas by African refugees and immigrants. The LBRF outbreaks in the Mediterranean and the Middle East regions were assumed to be extensions of epidemics from Africa, where *B. recurrentis* is proposed to have originated from *B. duttonii*. However, identification of *B*. *duttonii* ecotypes from soft ticks and relapsing fever patients in other geographical areas, e.g. Iran may suggest other likely origins for LBRF outbreaks.

The body lice may be a potential vector for re-emerging pathogens like *Yersinia pestis*. The archaic epidemiological evidence of the plague outbreaks has brought human body lice into attention as a competent vector of *Y. pestis*. The co-infection of *B. quintana* and *Y. pestis* in the dental pulp of skeletons excavated from a mass grave belonging to 11^th^ to 15^th^ centuries in Bondy, France proposed a louse-associated transmission of the plague in inland Europe at the medieval time.

Despite high standards of hygiene and preventive measures, human lice remain in our company, even in privileged countries such as France, Netherlands and United States. Recently, *Acinetobacter baumannii* has been detected in body lice from homeless people in different parts of the world. This bacterium has emerged as a highly troublesome pathogen due to its immense ability to acquire antibiotic drug resistance determinants. Therefore, we should always keep an eye on these companions. Strict survey of louse-borne diseases and the implementation of efficient delousing strategies should be public health priorities, especially in conditions of overcrowding or social disruptions.

## More details in:

1. ***High diversity and rapid diversification in the head louse, Pediculus humanus (Pediculidae: Phthiraptera)*.** M Ashfaq et al. 2015. Sci Rep; Vol. 5, 14188.
2. ***The genome of Borrelia recurrentis, the agent of deadly louse-borne relapsing fever, is a degraded subset of tick-borne Borrelia duttonii*.** M Lescot et al. 2008. PLoS Genet; Vol. 4, e1000185.
3. ***Evolutionary history of mammalian sucking lice (Phthiraptera: Anoplura)*.** J Light et al. 2010. BMC Evol Biol; Vol. 10, 292.
4. ***Phylogenetic analysis of the spirochete Borrelia microti, a potential agent of relapsing fever in Iran*.** S Naddaf et al. 2012. J Clin Microbiol; Vol. 50, 2873-2876.
5. ***Relapsing fever causative agent in Southern Iran is a closely related species to East African borreliae*.** S NAddaf et al. 2017. Ticks Tick Borne Dis; Vol. 8, 882-886.
6. ***Japanese Macaques (Macaca fuscata) as Natural Reservoir of Bartonella quintana*.** SH Sato et al. 2015. Emerg Infect Dis; Vol. 21, 2168-2170.
7. ***Origin of clothing lice indicates early clothing use by anatomically modern humans in Africa*.** MA Toups et al. 2011. Mol Biol Evol; Vol. 28, 29-32.
8. ***Apes, lice and prehistory*.** RA Weiss. 2009. J Biol; Vol. 8, 20.
9. ***Of lice and men: An itchy history*.** E Willingham. 2011. https://blogs.scientificamerican.com/guest-blog/of-lice-and-men-an-itchy-history/ (last accessed 28 April 2018).
10. ***Detection of Acinetobacter baumannii in human head and body lice from Ethiopia and identification of new genotypes*.** M Kempf et al. 2012. Int J Infect Dis; Vol. 16, e680-e683.

